# Direct observation of the conformational states of formin mDia1 at actin filament barbed ends and along the filament

**DOI:** 10.1091/mbc.E22-10-0472

**Published:** 2022-12-15

**Authors:** Julien Maufront, Bérengère Guichard, Lu-Yan Cao, Aurélie Di Cicco, Antoine Jégou, Guillaume Romet-Lemonne, Aurélie Bertin

**Affiliations:** a Institut Curie, Université PSL, Sorbonne Université, CNRS UMR 168, Laboratoire Physico Chimie Curie,75005 Paris, France; b Université Paris Cité, CNRS, Institut Jacques Monod, F-75013 Paris, France; CEA Grenoble

## Abstract

The fine regulation of actin polymerization is essential to control cell motility and architecture and to perform essential cellular functions. Formins are key regulators of actin filament assembly, known to processively elongate filament barbed ends and increase their polymerization rate. Different models have been extrapolated to describe the molecular mechanism governing the processive motion of formin FH2 domains at polymerizing barbed ends. Using negative stain electron microscopy, we directly identified for the first time two conformations of the mDia1 formin FH2 domains in interaction with the barbed ends of actin filaments. These conformations agree with the speculated open and closed conformations of the “stair-stepping” model. We observed the FH2 dimers to be in the open conformation for 79% of the data, interacting with the two terminal actin subunits of the barbed end while they interact with three actin subunits in the closed conformation. In addition, we identified and characterized the structure of single FH2 dimers encircling the core of actin filaments, and reveal their ability to spontaneously depart from barbed ends.

## INTRODUCTION

The actin cytoskeleton is essential to ensure cell motility, to regulate cell shape and architecture, and to control membrane reshaping during cell division (Mattila and Lappalainen, 2008; Dominguez and Holmes, 2011; Tojkander *et al.*, 2012; Blanchoin *et al.*, 2014). A myriad of actin-binding proteins ensures the fine regulation of the assembly and disassembly of actin filaments (dos Remedios *et al.*, 2003; Pollard, 2016). A large family of dimeric proteins called formins can track filament barbed ends to control their growth (Romero *et al.*, 2004). Formins play a crucial role in the generation of filaments found in stress fibers, filopodia or lamellipodia (Yang *et al.*, 2007).

Most formins gather several functional domains: a RhoGTPase-binding domain (GBD), a diaphanous inhibitory domain (DID), a dimerization domain (DD), two central formin homology domains (FH1 and FH2), and a diaphanous autoinhibitory domain (DAD). FH1 and FH2 are ubiquitous and are key to ensuring the main functions attributed to formins. The FH2 domains dimerize in a head-to-tail manner and encircle filament barbed ends. A “post” domain is located at the C-terminal end of each FH2 domain, while a “knob,” a flexible “linker,” and a “lasso” domain are located toward its N-terminus. The “lasso” domain of a given FH2 interacts with the “post” domain of its counterpart to induce FH2 dimerization. The “linker” domain is flexible and was shown to be either unstructured or able to adopt different secondary structures such as α-helices or β sheets (Higgs, 2005; Breitsprecher and Goode, 2013). Residues from both “post” and “knob” domains are engaged specifically in direct interaction with actin subunits at the barbed end (Higgs, 2005; Goode and Eck, 2007; Thompson *et al.*, 2013). FH1 domains are viewed as semiflexible chains of polyproline-rich domains that can recruit one or several profilin and profilin–actin complexes and tune barbed-end elongation rate (Paul and Pollard, 2008).

The molecular mechanism governing the tracking of the actin barbed ends by the FH2 domains remains elusive. Two antagonistic models have emerged to describe formin processivity at barbed ends, based on indirect evidence from x-ray crystallography of formin–actin complexes in nonnative states (Otomo *et al.*, 2005), from biophysical assays (Jégou *et al.*, 2013), and from molecular dynamics computations (Aydin *et al.*, 2018). Otomo *et al.* (2005) have generated the x-ray structure of a pseudoactin filament (with a 180° helical twist instead of the canonical 167°) bound to FH2 domains of yeast formin Bni1p, which are wrapped around the actin polymer in a continuous helix. In this nonnative structure, two actin-binding regions (ABR) could be identified within each FH2 domain, the “post/lasso” and the “knob” domains. A two-state “stair-stepping” model was proposed (Otomo *et al*., 2005). In this model, hypothetical conformational reorganizations of the FH2 domains take place around this structure where the FH2 dimer alternates between a closed and an open state. The closed state is derived from the observed structure by reorienting the linkers to form an FH2 dimer. In this state, the FH2 dimer would interact with three actin subunits simultaneously, and a steric clash from the leading FH2 would block monomer addition at the filament barbed end beyond the third terminal subunit. To allow the addition of an actin monomer, the Otomo and colleagues proposed an open state, where the FH2 dimer would interact with only the two terminal actin subunits at the barbed end, leaving the “post/lasso” actin-binding interface of the leading FH2 domain free and accessible. In this “stair-stepping” two-state model, it is hypothesized that, while one FH2 domain is bound to the actin filament via its two ABRs, the other FH2 domain undergoes a dynamic translocation, from one location where its two ABRs are bound to the filament (closed state) to another location closer to the barbed end with only one ABR bound (open state). After the addition of one actin subunit, the roles of the two FH2 domains are exchanged and formins cycle through these steps to control the elongation of filament barbed ends. This two-step model was used to fit the acceleration of elongation observed when pulling on a filament growing from a surface-attached formin (Jégou *et al.*, 2013; Cao *et al.*, 2018) and to account for the steps observed during formin-assisted elongation thanks to an optical trap (Kubota *et al.*, 2017).

Paul and Pollard (2008, 2009) have proposed an alternative model, named “stepping-second” because the translocation of the FH2 dimer, the “stepping,” is supposedly triggered by the incorporation of a novel actin subunit. In that model, the FH2 domains remain in the direct vicinity of the three terminal actin subunits at the barbed end, encircling them continuously. The terminal actin subunits are in rapid equilibrium between a closed conformation, with a helical twist near 180°, and an open conformation, with a more canonical 167° helical twist. These conformational changes are accompanied by FH2–actin contact modulations and by the stretching of the FH2 linkers. Upon addition of a new actin monomer, the canonical 167° helical twist would be consolidated, thereby straining durably the FH2 linkers. The translocation of one FH2 domain toward the barbed end would relax this strain. After translocation, the barbedend helical twist would again be in rapid equilibrium between 167° and 180°.

**FIGURE 1:**
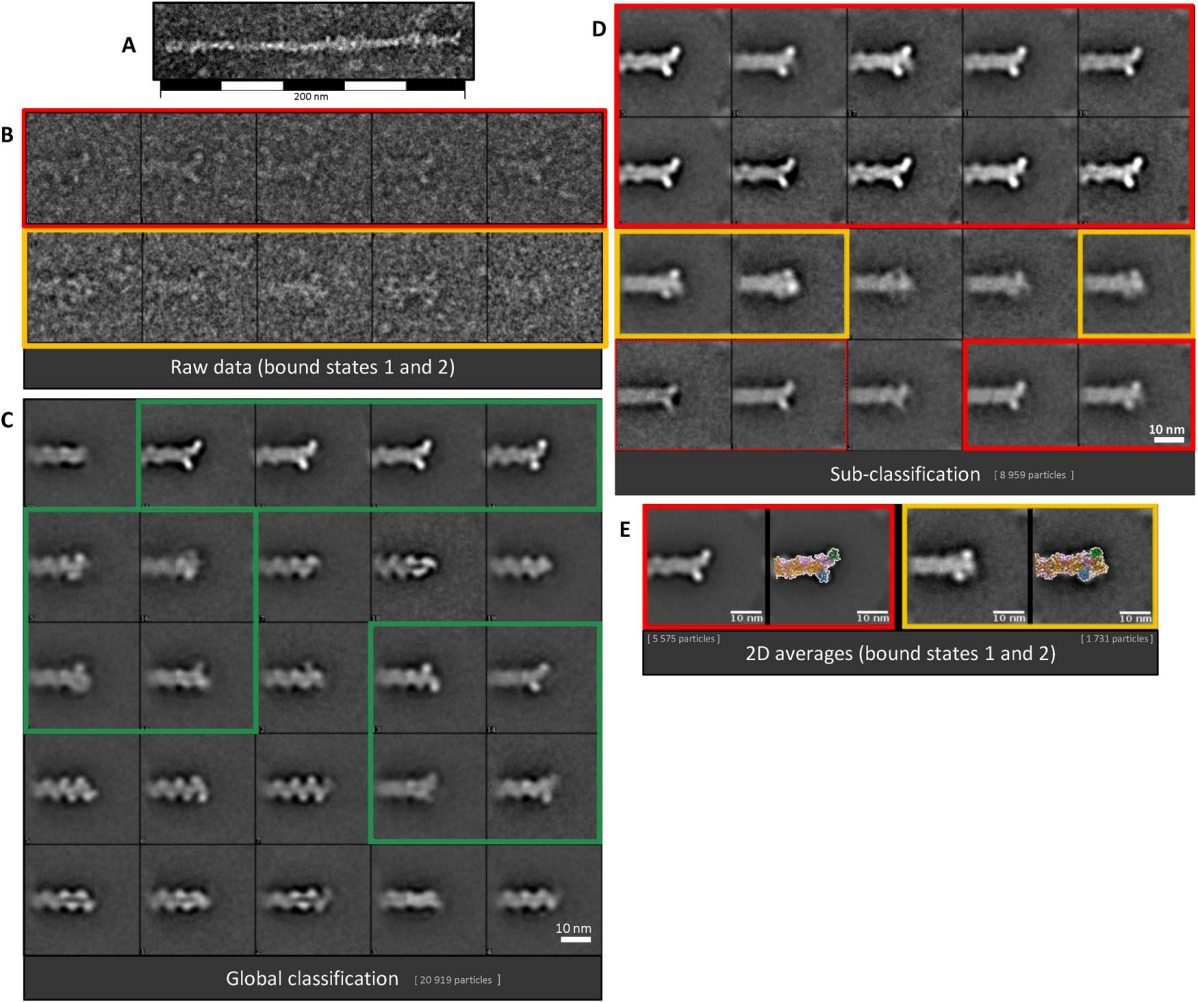
2D patterns observed at actin filament ends in the presence of formins. (A) Image of an actin filament end, displaying an extra density. (B) Raw images corresponding to the majority (top row, red window) or the minority (bottom row, orange window) 2D classes of formin-bound actin filament barbed ends. (C) SPIDER 2D classes generated from actin filament ends (20,922) observed by negative staining EM after actin filament sonication, followed by incubation with 100 nM mDia1 formins. Green windows: 2D classes of actin filament ends showing additional densities attributable to bound formins. (D) 2D classes generated from a subselection of actin filament ends, formin-bound candidates (C, green windows). Red windows: majority 2D classes showing additional densities protruding from the actin filament tip. Orange windows: minority 2D classes showing additional densities embedded at the actin filament tip. (D) Global class averages of the majority (red window, 3804 particles) or the minority (orange window, 907 particles) configurations observed alone (left) or overlaid with the atomic models of a barbed end interacting with a formin (right) in the open (red window) or the closed (orange window) state according to the “stair-stepping” model (Otomo *et al*., 2005). (E) 2D averages of open (highlighted in red) and closed (highlighted in orange) states, where crystal structures (PDB 1Y64 and 5OOE) of actin and formins are superimposed.

**FIGURE 2:**
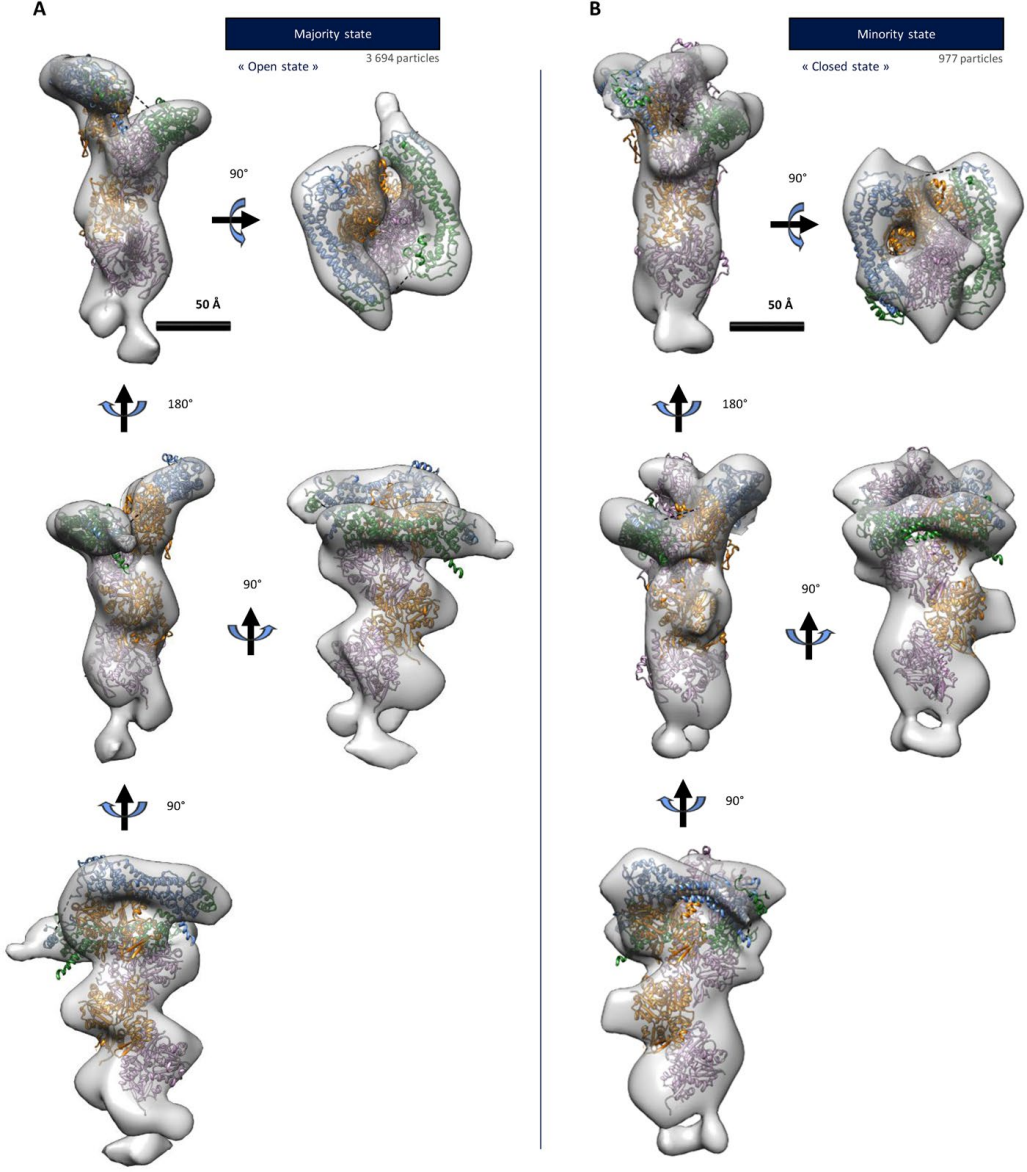
3D structures of formin-bound barbed ends. RELION 3D reconstructions of the majority configuration (A, 2048 particles, 29 Å resolution) and the minority configuration (B, 402 particles, 32 Å resolution) of the formin-bound barbed ends (gray) in which an atomic structure of the open (A) or the closed (B) states described by the “stair-stepping” model are fitted (PDB 1Y64/5OOE). Green/blue: FH2 domains. Orange/pink*:* actin subunits.

**FIGURE 3:**
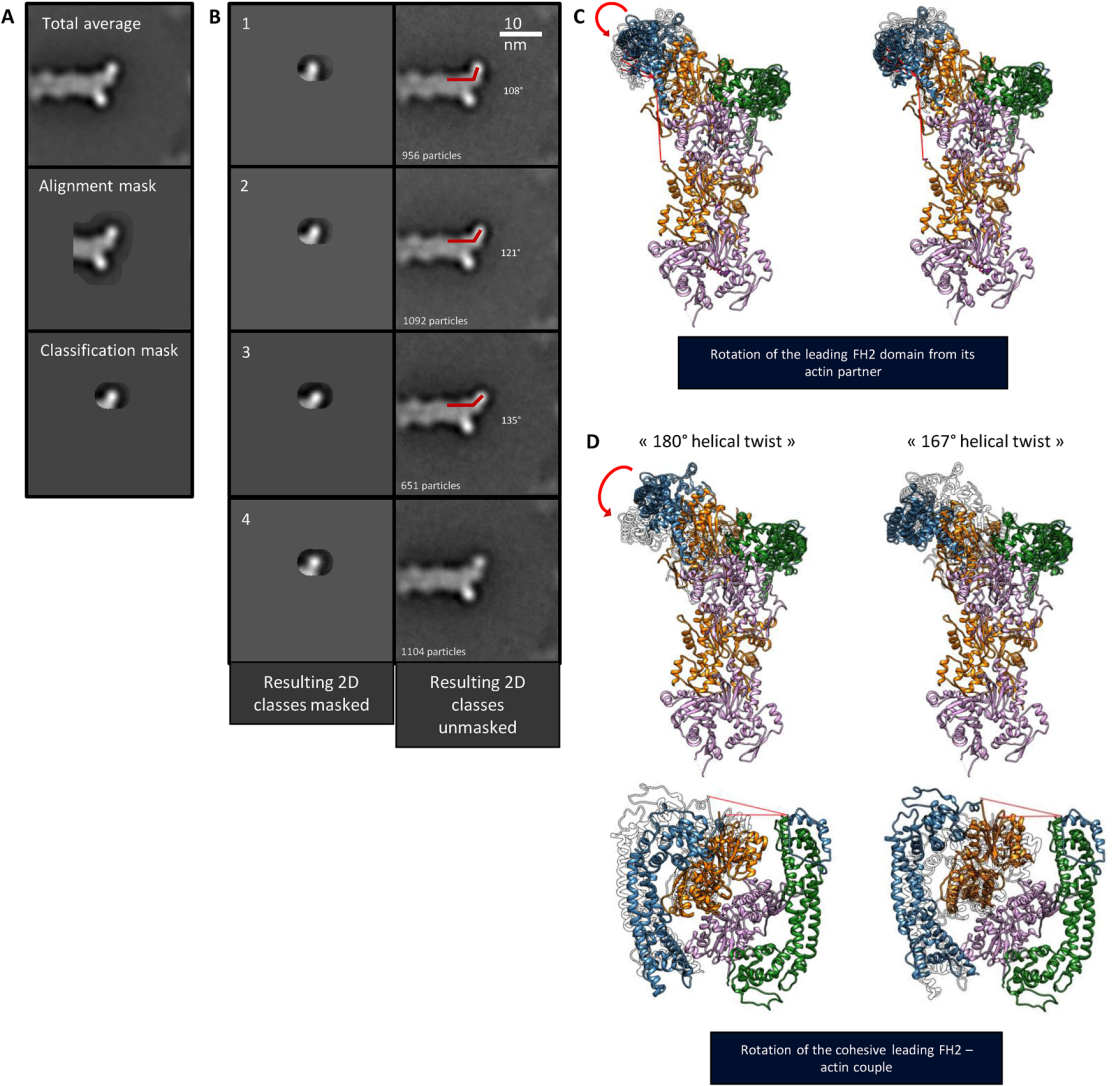
Structural variations in the open state revealed by focused 2D analysis with two hypothetical corresponding 3D models. (A) Top: total average of formin-bound barbed ends in the open state. Middle: total average displayed through the mask used for particle alignment. Bottom: total average displayed through the focused mask used for particles classification. (B) Left: classes generated after focused classification and displayed through the focused mask used. Right: classes generated after focused classification and observed without masking. (C) Atomic models of a barbed end capped with a formin in the open state derived from the previously determined arrangement (see Figure 2A) and presented after an independent rotation of the leading FH2 domain in the trigonometric (left) or antitrigonometric (right) direction. For each two positions shown, the opposite FH2 position is displayed in transparency. (D) Atomic model of a barbed end capped with a formin in the open state (left) and presented in the previously determined arrangement (see Figure 2A) or after reorientation of the first actin subunit (orange; top right). Top: side view. Bottom: top view. Green/blue: FH2 domains. Orange/pink: actin subunits. (C, D) The red bent arrows describe the rotation movement behavior of the leading FH2 domains, while the straight red lines describe materialized angles formed by the filament axis and the FH2 orientation in different configurations.

**FIGURE 4:**
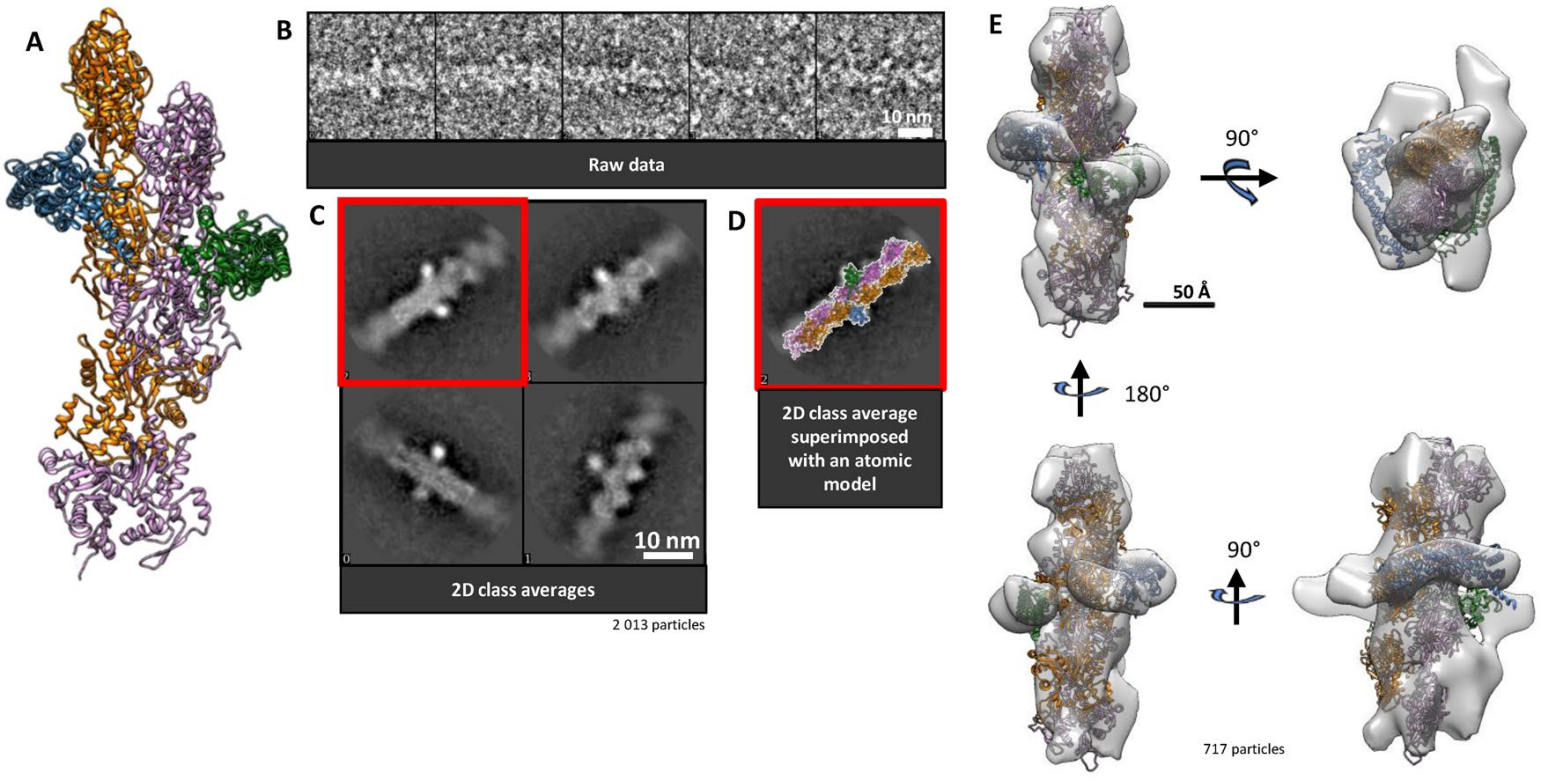
Formin interaction mode with actin filament by encircling the helical body. (A) Atomic model of an FH2 dimer encircling an actin filament “slightly” away from the barbed-end location. This atomic model was constructed according to the open state of the stair-stepping model where the leading FH2 domain has been moved away from the filament axis (see Figure 3) and two actin subunits have been added at the barbed end. (B) Raw images of particles identified as FH2 domains encircling the actin filament. (C) RELION 2D classes generated from particles picked along actin filaments previously sonicated and incubated with formins. (D) Class average (from C. red window) matched with the 2D pattern of an FH2 dimer encircling the actin filament body. (E) 3D reconstruction of a formin encircling the actin filament body (717 particles) in which atomic structures are fitted (PDB 1Y64/5OOE). Green/blue: FH2 domains. Orange/pink: actin subunits.

The primary purpose of our investigations was to discriminate between these two models by using electron microscopy (EM) to directly observe the conformations of the formin mDia1 FH2 dimer at the filament barbed end. To achieve this, we have optimized the sample preparation to enhance the density of single short filaments (Supplemental Figure 1) and adapted the image processing workflow to the processing of our filamentous ends. Indeed, only a few reports describe the structure of proteins at either the barbed or the pointed ends of actin filaments, and they involve proteins that stably cap the ends and thus neutralize the actin filament dynamics (Narita *et al.*, 2006; Shaaban *et al.*, 2020; Funk *et al.*, 2021). We report here the direct observation in negative stain EM of FH2 dimers with elongating actin filament barbed ends (Figure 1). Using negative stain EM allows us to identify two major conformations (Figures 2 and 3) that argue in favor of the “stair-stepping” model. Furthermore, we report a conformational variability of the open state (Figure 3). We also resolved FH2 dimers in interaction with the core of the filament (Figure 4). This indicates that formins can find themselves away from the barbed end more frequently than previously thought. Those findings should be key for the actin field and prompt future investigations.

## RESULTS

To observe formins at actin filament barbed ends, we first optimized the density of short actin filaments and thus the number of actin ends adsorbed onto an EM grid, as described in Supplemental Figure 1 and in *Materials and Methods*. Sonication was carried out on 1 μM rabbit α-skeletal preformed actin filaments in F-buffer at pH 7.8 with 50 mM KCl in order to shorten them before exposing them to formin mDia1 (see *Materials and Methods*). Sonication has been reported to induce the depolymerization of actin filaments (F-actin), in addition to their severing (Carlier *et al.*, 1985). To quantify the amount of actin monomers (G-actin) generated by our sonication step, we performed measurements using pyrene-labeled actin (Supplemental Figure 2) with samples prepared the same way as for EM. They allow us to determine that, when we fixated our samples for EM observation, there was 250–350 nM G-actin in solution, and thus mDia1-bearing barbed ends were elongating at a rate of 1–2 subunits/s.

### Two-dimensional single particle image processing of actin barbed ends

As compared with single particle analysis (SPA) performed on globular proteins, our objects of interest are circumscribed to the ends of elongated asymmetrical assemblies whose upstream bodies extend beyond the periphery of the extracted boxes. Raw boxed particles and an example of filamentous ends displaying an additional density are presented in Figure 1, A and B. We thus adapted SPA to our specific sample to optimize the analysis of the filament ends. The two-dimensional (2D) analysis is schematically summarized in Supplemental Figure 3 and presented in *Materials and Methods*. After a first round of the alignment protocol, a 2D classification was performed to select the particles corresponding to actin filaments interacting with formins. A second round of the same alignment protocol was performed again with the data set extracted from the classes displaying a specific signal at the actin ends (highlighted in green in Figure 1B). At this stage, an additional 2D classification was carried out to improve the resulting 2D classes.

From the resulting 2D analysis, three distinct groups can be identified. From the first round of classification, most of the generated classes correspond to bare actin filament ends (Figure 1C). These classes are identical to the classes obtained for an actin control sample without formins (see Supplemental Figure 4). These strictly naked actin filament ends represent ∼57% of the data set (11,960 out of a total of 20,919 particles). These classes were not considered for the second round of classification. From a second round of classification for filaments potentially displaying formins (Figure 1C, green boxes), two additional groups (see Figure 1, D and E, red and orange highlights) clearly display additional densities with determined shapes at the actin filament ends. The classes highlighted in red show a stereotypical “Y shape” and represent 26% of the total data set if we consider only averages with enough contrast and defined details (Figure 1D, continuous red boxes, 5575 out of 20,919) to 31% of the total data set if we include averages displaying blurry features (Figure 1D, continuous and dashed red boxes, 6356 out of 20,919). The data from the classes showing blurry features (5%) were not considered for further processing. The corresponding global average can be superimposed with a 2D projection of an atomic model structure of the open state of the “stair-stepping” model (Figure 1E, red box). Atomic models were built using previously published actin and formin FH2 crystallographic structures (Protein Data Bank [PDB]: 5OOE and PDB: 1Y64, respectively). The classes highlighted in orange are in minority, representing 8% of the total data set (1731 particles out of 20,919), and the corresponding global average can be superimposed in 2D with an atomic model structure of the closed state of the “stair-stepping” model (Figure 1E). The proportion of ends in the open state among all the ends where a formin can be visualized adds up to 76–79%, depending on whether one considers averages with or without blurry features. To go further, the aligned particles belonging to the classes corresponding to either the open or the closed state, and displaying enough contrast, were merged to be finally subjected to 3D classifications.

### Open and closed conformations of FH2 mDia1

To characterize the possible different conformations in 3D, we have performed multireference 3D classifications and reconstructions merging the two sets of particles displaying additional density at actin filament ends within the 2D analysis (see Figure 1, red and orange boxes, 7306 particles). As initial references, high-resolution structures of a bare actin filament barbed end (PDB: 5OOE) and open and closed states of the “stair-stepping” model (generated using PDB: 1Y64) were selected and filtered to low resolution (50 Å). We used the crystal structure from yeast Bni1p FH2 domains in interactions with an actin filament, rather than the existing one from the mammalian mDia1 formin FH2 dimer in isolation (PDB 1V9D), because actin–formin contacts are described in the Bni1p structure. Three 3D classes were obtained during the first round of 3D classification. Two classes reflect two distinct conformations of formin bound to the barbed end of actin filaments. A third class corresponds to misaligned particles. The sub–data set corresponding to the first two 3D classes were independently subjected to further 3D classification followed by 3D refinement and postprocessing. The resulting 3D reconstructions are shown in Figure 2, A and B, gathering, respectively, 3694 (18%) and 977 (5% of the total data set) particles. They reach a resolution of 26 and 27 Å, respectively (Supplemental Figure 9). The first class gathers 79% of formin-bound filament barbed ends identified by 3D classification, while the second class gathers 21% of these formin-decorated barbed ends.

**FIGURE 5:**
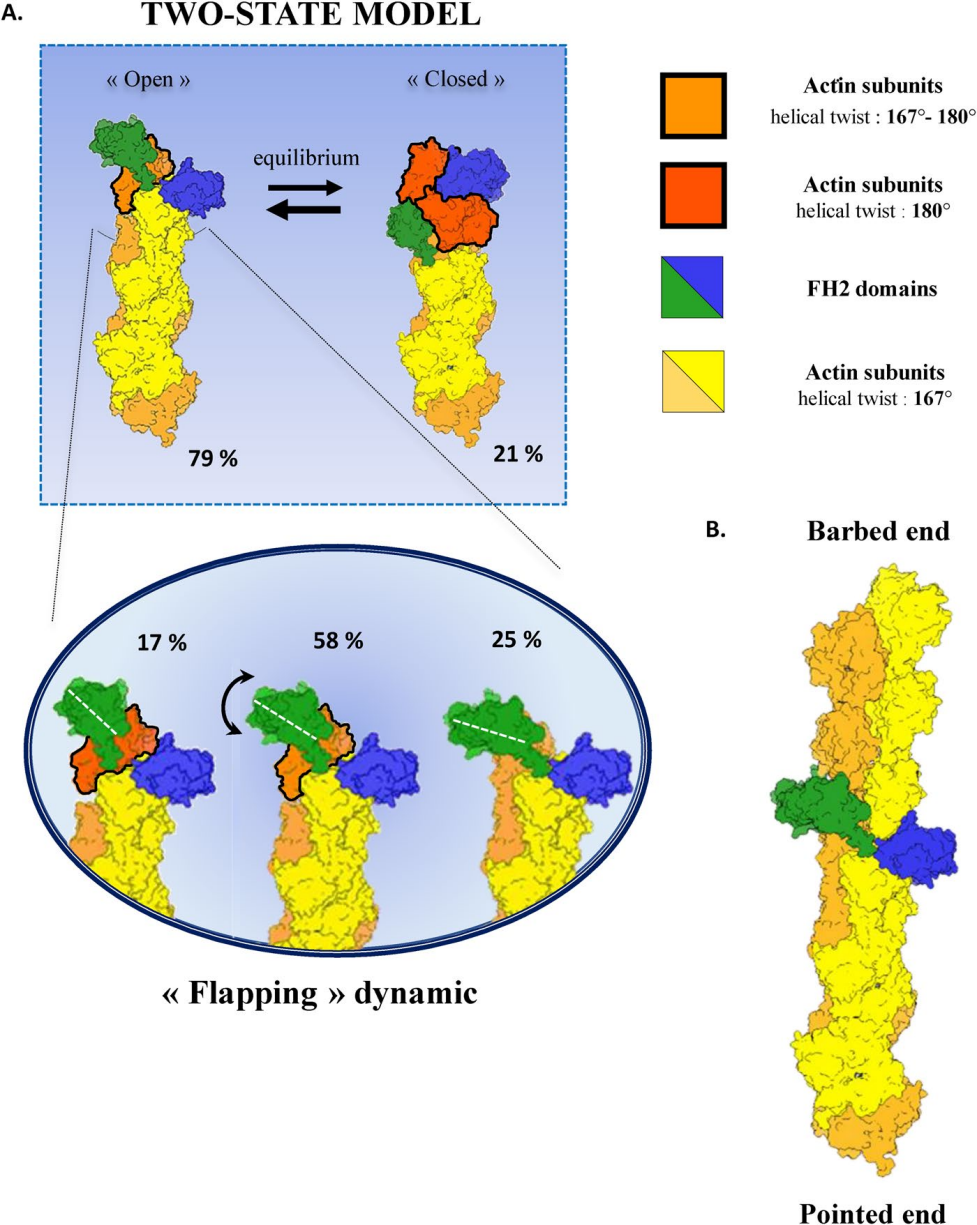
Schematic representation of formin FH2 domains bound to actin. (A) Top: schematic representation, based on our 3D reconstructions, of the two-state model proposed by the stair-stepping model with percentages indicating the relative distributions of the open and closed states as determined in Otomo *et al.* (2005). Bottom: schematic representation of the flexibility observed in the open state, with the relative distributions of the main substates with percentages indicating the distribution between the main substates. The two strands of the long-pitch double helix are shown in two colors, orange and yellow, for readability. Each of these subunits makes a 167° angle with its nearest short-pitch neighbor. Actin subunits at the barbed end displaying an angle close to 180°, with respect to their short-pitch neighbor, are shown in red. Actin subunits at the barbed end displaying an intermediate angle, between 167° and 180°, with respect to their short-pitch neighbor, are shown in dark orange. The FH2 domains are shown in green or blue. The color code is displayed on the right-hand side of the scheme. (B) Schematic representation of FH2 domains encircling the core of an actin filament. The color code is identical to that in A.

Within these 3D envelopes, a segment of actin filament extends toward the pointed end as already shown by the 2D classes. At the barbed ends, some additional density can be visualized that displays the shape of an FH2 formin dimer. To confirm and clearly delineate the nature and relative orientation of the proteins within our 3D classes, we have carried out automated rigid docking of high-resolution structures in our envelope. The PDB 1Y64 crystallographic structure (Otomo *et al.*, 2005) of an actin subunit in complex with a single Bni1p FH2 formin domain was docked into the 3D structures. Superimposed crystal structures of Bni1p and mDia1 FH2 dimers, docked in our envelope (Supplemental Figure 10) revealed minor differences except at the formin–actin contact sites. This finding further convinced us that using a crystal structure of a formin in interaction with actin was more relevant. The “Fit in Map” tool from Chimera (Pettersen *et al.*, 2004) was used to dock a doublet of the actin–formin complex. In this high-resolution starting model, the actin subunits bound to FH2 domains were set at a helical twist of 180°. Two additional actin subunits from the PDB 5OOE structure were docked toward the pointed end, with a canonical helical twist of 167° (Merino *et al*., 2018). Following this “rigid” global docking, a local adjustment was performed by a “sequential fit” with Chimera to finely dock the FH2 domains independently from one another. The helical twist of the actin subunits toward the pointed ends and the actin–FH2 (“knob”) contacts are imposed and static in the docking process. The resolution of the envelope obtained following this procedure does not allow one to decipher the helical twist adopted by the two terminal actin subunits at the barbed end. Nevertheless, the first 3D class (Figure 2A) representative of most of the particles (79% of formin-bound filament barbed ends) closely matches the open state described by Otomo *et al.* (2005). Indeed, the FH2 domains, highlighted in blue and green, in Figure 2, are bound to only two actin subunits and unambiguously show one free and accessible “post” domain that could bind a third actin subunit. The fact that this conformation corresponds to 79% of the identified barbed ends with a bound formin strongly argues in favor of the two-state “stair-stepping” model (Figure 5, top left-hand corner), as a conformation where the post domain of the leading FH2 is not interacting with any actin subunits does not exist in the “stepping-second” model (Paul and Pollard, 2008). The second 3D class (Figure 2B) contains an additional actin subunit protruding from the FH2 dimer at the barbed end. In this conformation, the FH2 domains encircle three actin subunits simultaneously, matching the closed state of the “stair-stepping” model.

To ensure that formins were also visible at the barbed ends of actin filaments in a more physiological condition and were not artifacts from staining and drying by negative stain EM, we carried out cryo-EM experiments. To enhance the density of filaments on the cryo-grids, we used C-flat grids coated with a layer of graphene as described by Palovcak *et al.* (2018) (see *Materials and Methods*). After sample cryo-fixation, we collected data using a 200 kV field emission gun (FEG) Glacios microscope. Typical images are displayed in Supplemental Figure 5. From 3405 images, 12,112 ends were manually picked (See Supplemental Figure 6A). Given that the signal-to-noise ratio and the density of ends are lower for cryo-EM images, the final resolution obtained from these data was unfortunately not better than what we obtained with negative stain images. Nonetheless, in 2D classes, additional densities can be pinpointed at the ends of filaments (see Supplemental Figure 6B). Owing to the limited number of ends observed, we identified primarily one conformation of formin bound to the barbed ends. From 3D reconstruction and classification, one main structure was generated (Supplemental Figure 6C). Actin subunits as well as formin FH2 domains were docked within this structure. The FH2 dimers connect only the two terminal actin subunits of the barbed end with a free and accessible post domain, a recognizable feature of the open conformation.

### Conformational flexibility of the open conformation

After the generation of 2D classes, we noticed that the open-state class (see Figure 1, red) displayed some striking variability in the orientation of the FH2 domains with respect to the actin filament. To further analyze this variability, we applied a classification mask centered on the leading FH2 domain (see Figure 3A). Using this mask, the particles found to be in the open conformation (3804 particles) underwent a classification procedure. The orientation of the leading FH2 domain unambiguously varies as shown in Figure 3B. The amplitude of the angular fluctuation was assessed using ImageJ (see *Materials and Methods* and Supplemental Figure 7). Four classes were obtained displaying three different orientations of the leading FH2 domain (Figure 3, B and C). In class 1 (25% of the particles), the angle between the actin filament long axis and the leading FH2 domain is the smallest (108°). In classes 2 (29% of the particles) and 4 (29% of the particles) similar intermediate orientations of the FH2 domains (121°) are found. The details featured in class 4 displayed a lower signal-to-noise ratio and thus looked noisier than in class 2, suggesting that within class 4 the conformation slightly varies around the 121° orientation. In class 3 (17% of the particles), the angle between the leading FH2 domain and the actin filament axis is the most obtuse (135°). The amplitude of the fluctuations likely reflects the existence of a continuum of conformations. Indeed, tuning the threshold for classification can generate more classes whose orientations only slightly differ from one another. We chose four classes to make the conformational change clearly visible. In those 2D classes, the actin filament holds the same orientation. The analyzed variability therefore does not result from different views and orientations of the sample. Moreover, the Euler angle distribution assigned during the previously shown 3D reconstruction was analyzed regarding the different 2D classes shown here and their belonging particles. No specific orientation trend could be discerned within the observed 2D classes. Given the small number of particles combined per 2D class and the hypothesized underlying conformational continuum, the generation of 3D structures for these intermediate conformations was not considered.

### FH2 domains encircling the actin filament body

Using single-molecule fluorescence microscopy, it was previously shown that mDia1 formins can be displaced from actin filament ends by a capping protein, and formins were then observed to perform 1D diffusion along the actin filament core (Bombardier *et al.*, 2015). Within our images obtained in the absence of capping proteins, we could indeed pinpoint additional densities alongside actin filaments (see Figure 4B). We started our analysis with 2013 extra densities manually picked and subjected to a 2D classification. The 2D classes are presented in Figure 4C. Additional densities on the side of the filaments are highlighted. The superimposition of high-resolution FH2 and actin structures onto one of the 2D classes (Figure 4D) suggests that they are indeed FH2 domains. The particles from the last class (717 particles) were used to generate a 3D structure (Figure 4E). Some distortion, visible on the 3D reconstruction, resulted from a predominance of views and thus of available angles in the 3D reconstruction. Indeed, automated particle picking was unsuccessful and manual picking induced the selection of preferential views.

Nonetheless, we reconstructed a 30 Å resolution structure where the FH2 domains as well as the actin subunits can be pinpointed and on which an FH2 dimer can be docked encircling an actin filament. The low resolution of the reconstruction is sufficient to show that, alongside actin filaments, the relative arrangement of the FH2 domains and actin is comparable to the ones observed at the actin barbed ends. Our observations thus suggest that FH2 dimers can diffuse along actin filaments by interacting with specific binding sites. We estimated that we could identify on average ∼1 formin per 4 μm of actin filament and that ∼8% of the observed filaments had a formin along their core (*Materials and Methods*; Supplemental Figure 8).

## DISCUSSION

### Direct observation of formin–actin interaction by negative stain electron microscopy

Using EM, we have, for the first time, directly imaged mDia1 FH2 dimers in interaction with polymerizing actin filaments. The medium-resolution conformations obtained in this study allow us to discriminate between two previously proposed models (Otomo *et al.*, 2005; Paul and Pollard, 2008) describing the conformations of formins interacting with growing barbed ends, in favor of the stair-stepping model. Indeed, we clearly observe a conformation where one FH2 dimer interacts with only two actin subunits and where the leading FH2 domain partly hangs in solution in front of the barbed end. This conformation is predicted by the stair-stepping model and is completely absent from the stepping-second model.

Nevertheless, our structures have a limited resolution so that finer structural details remain to be uncovered. For instance, we could not directly determine whether the actin subunits of the barbed ends are rather organized with a canonical 167° or a nonstandard 180° helical twist as suggested earlier Otomo *et al.* (2005). In Otomo *et al.* (2005), the 180° noncanonical helical twist observed in the crystal might result from the presence of FH2 bound in a “daisy-chain” manner to all of the actin subunits along the filament. It would also be sensible, in a future investigation, to determine actin helicity with formins bound alongside the core of actin filaments.

Compared to recent publications describing bound pointed ends (Shaaban *et al.*, 2020) or barbed ends at higher resolution (Funk *et al.*, 2021), the dynamic nature of formins at the barbed end makes the production of a high density of short filaments very challenging. In these aforementioned studies, the capping protein CapZ that has been used restricts actin polymerization and thereby permits the production of a high density of short capped filaments that can be isolated through size exclusion chromatography and subsequently further concentrated (Funk *et al.*, 2021). In our case, to obtain a high density of short filaments without disturbing the dynamic at the barbed end and in addition to sonication, the use of additional actin-binding proteins either to sequester monomeric actin in solution or to enhance actin nucleation (see Supplemental Figure 2C) did not lead to any significant improvement of the quality of the data. Our attempts at using additional proteins to trap G-actin (Gc-globulin, 300 nM; Supplemental Figure 2C) or enhance filament density (spectrin–actin seeds, 150 pM) did not provide a higher density of formin-bearing barbed ends per field of view (Supplemental Figure 2C). Besides, the presence of additional proteins induced noise preventing proper image analysis, and our main interest was to describe the conformations of formins at the barbed end of actin filaments. Sonication, our most efficient strategy, was not efficient enough to produce a higher barbed-end density that would lead to higher-resolution structures.

### Occupancy rate of the open versus closed state

A widely used characteristic of formins is their so-called “gating factor.” It is defined as the ratio between the elongation rate of formin-bearing and the elongation rate of bare barbed ends, in the absence of profilin. Among Formins, mDia1 has a notably high gating factor of 0.9. The gating factor is often assumed to represent the fraction of time that the formin spends in the open state. However, this assumption relies on the hypothesis that the monomer on-rate is the same for a bare barbed end as for a formin-bearing barbed end in the open state, which appears very unlikely. Kinetic measurements thus provide limited insights into the relative time spent by a formin in open and closed states.

Alternatively, in an earlier study (Jégou *et al.*, 2013), by measuring the elongation rate of mDia1-bearing barbed ends as a function of mechanical tension, we estimated that the FH2 dimer was in the open state 56% of the time, in the absence of tension. This estimation is lower than the outcome of the structural assay presented here (79%). This estimation relies on the consideration that the applied tension skews the state occupancy in favor of the open state, and the experimental data were fitted based on the hypothesis that tension is applied equally to both FH2 hemidimers. With an alternative analysis, considering that the force is applied to only one of the two FH2 hemi-dimers (see Supplemental Text), we would obtain an even lower estimation (12%) of the open-state occupancy rate in the absence of tension. In these former experiments, filaments were elongated in the presence of profilin–actin, which binds to polyproline tracks of the FH1 domains to deliver actin to the barbed end. The simultaneous interaction of profilin–actin with the FH1 domain, and with the FH2-bound barbed end, transiently forms a “ring” complex (Vavylonis *et al.*, 2006; Cao *et al.*, 2018). One cannot exclude that the formation of this ring complex might decrease the open-state occupancy rate, compared with the absence of profilin.

Our direct assessment of the open-state occupancy rate thus provides important information on the molecular nature of the formin–barbed end conformations that could not be directly inferred from kinetic measurements, with or without mechanical tension, so far. Considering a gating factor of 0.9 and considering that formin mDia1 spends 79% of the time in the open state, we can compute that the on-rate for monomers would be slightly higher (14% higher) for an mDia1-bearing barbed end in the open state than for a bare barbed end. We hypothesize that the available actin-binding interface on the leading FH2 domain could provide a first docking intermediate for actin monomers that would help their orientation relative to the barbed end, resulting in a higher on-rate.

We also reveal that, in the open state, the orientation of the leading FH2 relative to the long axis of the filament fluctuates (Figure 3). Several conformations can be distinguished, suggesting a continuum of conformations. These conformations are likely to have different on-rate constants for the addition of actin subunits at the barbed end. This could partially explain why previous measurements, based on assembly rates, estimated a lower occupancy rate for the open state (Jégou *et al.*, 2013).

### Flexibility of the open state: “flapping” model

We propose a “flapping” model where the angle between an FH2 domain and actin varies from 108° to 135° (Figure 5). We designed extrapolated 3D models from existing high-resolution crystal structures that would exhibit, in 2D projections, the observed angular “flapping” fluctuations (Figure 3). In a first attempted model, one can examine whether the rotation of an FH2 domain independently from the filament barbed end structure would be possible. As shown in Figure 3C, the rotation is not constrained by any steric clash and would be allowed by the flexibility of the FH2 “linker” domain. However, upon rotation, the FH2 “knob” domain might not interact properly anymore with the terminal actin subunit. Hence, a second more favorable model is proposed (Figure 3D) where the terminal actin subunit would rotate with a slight bow and thus drag an FH2 interacting through its “knob” domain. In such a configuration (Figure 3D), the contacts between the FH2 domain and actin subunits would thus be preserved and their relative orientation and spacing imposed. Following this rearrangement, the actin helical twist would vary in a range included between the two observed values of 180° (structure 1Y64) and 167° (structure 5OOE) similarly to the “stepping-second” mechanism (Paul and Pollard, 2008) and to what was consequently assessed by molecular dynamic simulations (Aydin *et al.*, 2018). However, our data exclude the “stepping-second” model to describe FH2 dimer translocation in actin poly­merization. The terminal actin subunits at the barbed end would thus show a configuration closer to the helical twist angle found within canonical actin filaments, and the previously characterized formin–actin contacts in 1Y64 would be preserved. Nonetheless, while this angular switch cannot be directly observed within our medium-resolution structures, the proposed mechanism is a sensible hypothesis that would reflect the “flapping” FH2 domain motion toward its most open conformation. Indeed, the two actin filament arrangements considered as the extreme configurations of such a motion (PDB structures 1Y64 and 5OOE) differed not only by their helical twist value but also by the actin subunits’ orientations relative to the filament axis (Otomo *et al.*, 2005) resulting in the actin subunit rotating outward within the most open conformation. In this extreme open conformation, after the transition from an actin helical twist of 180° to 167°, an actin monomer (polymerization) or an actin oligomer (annealing) could be added at the barbed end in the canonical actin filament conformation (Supplemental Figure 11). Indeed, in our assay, actin filament annealing could occur, in addition to polymerization, following sonication.

### Formin FH2 dimers spontaneously translocate from the barbed end to the core of the filament

Our observation of formin mDia1 along the core of actin filaments (Figure 4; Supplemental Figure 12) is reminiscent of previous reports on other formins. In vivo observations, where budding yeast Bni1 (Buttery *et al.*, 2007) and fission yeast for 3p (Martin and Chang, 2006) formins displayed a retrograde mobility with actin cable elongation, were attributed to formins being bound to actin filaments and not actively elongating filament barbed ends. Gurel *et al.* (2014) obtained a 3D structure using high concentrations of mammalian INF2 formins and could see stacked or “daisy chain” FH2 domains decorating the core of actin filaments. On formin mDia1, another study (Kubota *et al.*, 2018) reported a stepwise translocation that ranged from one to two or three actin subunits away from the filament barbed end. More strikingly, Bombardier *et al.* (2015) showed that capping protein could displace formin mDia1 from the barbed end and consequently observed it diffusing along the core of the filament, micrometers away from the barbed end. Here, we observe mDia1 several to tens of subunits away from the barbed end, in the absence of capping protein to displace it from the barbed end.

The flapping of the FH2 domains of mDia1 that we described earlier could provide an explanation for this observation. In the extreme open conformation, the addition of actin subunits at the barbed end could leave the FH2 dimer “lagging” behind and no longer translocating synchronously with the elongating barbed end (Supplemental Figure 11A). Lagging formins would then start diffusing along the actin core, in both directions, as described by Bombardier *et al.* (2015).

Other scenarios could be proposed to account for the presence of formins along the core of actin filaments. We can imagine the following: a formin FH2 dimer could open up and encircle the core of a filament; the formin could be displaced from the barbed end by the arrival of another formin (playing the role of the capping protein in the mechanism proposed by Bombardier *et al.*, 2015); the formin-bearing barbed end could bind to the pointed end of another filament (annealing; Supplemental Figure 11B); finally, a formin could access the core of the filament via the filament pointed end.

In the lagging scenario, the formin going to the core of the filament leaves behind a bare barbed end that will have a limited time to bind another formin before the sample is fixated for observation. The lagging scenario is thus the only one predicting that filaments with a formin along their core should be less likely to also exhibit a formin at their barbed end, compared with the global filament population. To test this prediction, we randomly picked 38 micrographs in which we found 100 filaments with an observable end and with a formin along their core. Of these 100 filament ends, only 24 had a formin. Considering that half of the 100 ends were barbed ends, we can estimate that 48 ± 7% (*n* = 50) of actin filaments with a formin within their core also display a formin at their barbed ends. This is significantly less than for the global filament population, where 77 ± 0.4% (*n* = 10,461) of barbed ends are decorated with formins. This does not exclude the other scenarios, but it supports the lagging scenario as a likely mechanism putting formins along the core of the filament.

A possible consequence of the lagging mechanism is that formins would be more likely to lag behind and less likely to catch up, by diffusion, with a rapidly growing filament end. This would result in an apparent decrease of formin processivity with increasing elongation rates, in agreement with earlier in vitro observations (Paul and Pollard, 2008; Cao *et al.*, 2018). Future experiments are needed to test this possibility.

In vivo, several mechanisms may regulate the probability for a formin to lag behind filament barbed ends. First, the formin-assisted elongation of filament barbed ends is expected to occur primarily from profilin–actin complexes delivered to filament barbed ends by FH1 domains. This mode of elongation implies frequent interactions between FH1 and the barbed end (Vavylonis *et al.*, 2006; Cao *et al.*, 2018), which may likely reduce the probability of formins to lag behind the barbed end. Second, membrane anchoring of active formins (Suzuki *et al*., 2020) may restrict their ability to diffuse along the core of actin filaments.

In conclusion, our EM observations provide a direct visualization of the different conformational states of mDia1 formins interacting with actin filaments and refine the processive “stair-stepping” model proposed by Otomo *et al.* (2005), by showing a continuum of conformations for the open state. We report here that formins can unexpectedly be found within the core of actin filaments in the absence of capping proteins that would displace them from the barbed end. We expect that future studies will reveal finer details of the terminal actin subunit arrangement of formin-bearing barbed ends.

## MATERIALS AND METHODS

Request a protocol through *Bio-protocol*.

### Protein purification and storage

Actin was purified from rabbit muscle as detailed previously (Rubenstein and Spudich, 1977; Wioland *et al.*, 2017) and stored up to 4 weeks in G-buffer (5 mM Tris, pH 7.8, 0.2 mM ATP, 0.1 mM CaCl_2_, 1 mM dithiothreitol [DTT], and 0.01% NaN_3_) on ice.

Recombinant mouse mDia1(SNAP-FH1FH2-DAD-6xHis) formin (uniprot O08808, seq. 552–1255 aa) was expressed in *E. coli* and purified through immobilized metal affinity chromatography (HisTrap) followed by steric exclusion chromatography (HiLoad 16/60 Superdex 200) as developed in Jégou *et al.* (2013). Formins were snap frozen and stored at –80°C in 50 mM HEPES buffer, pH 7.8, 200 mM KCl, 10% glycerol, and 1 mM DTT.

### Pyrene assays

Actin (5 μM; 5% labeled with pyrene) was polymerized at room temperature for 1 h in F-Buffer (5 mM Tris-HCl, pH 7.8, 1 mM MgCl_2_, 0.2 mM EGTA (Tetra[acetoxymethyl Ester]), 0.2 mM ATP, 1 mM DTT, 50 mM KCl) to reach steady state. A total volume of 500 μl containing the polymerized F-actin diluted to 1 µM was sonicated in F-buffer. The sonication was operated with a sonicator Vibra-Cell 75041 (20 kHz, 750 W, 20% power) equipped with a 3-mm-diameter probe. Ten pulsations of 0.1 s separated by a 3.9 or a 4.9 s rest period were used for the sonication. Sonicated solution (200–300 μl) was then inserted into quartz cuvettes and analyzed for fluorescence signal for at least 20 min using a spectrofluorimeter (Safas; Xenius). The average time from the end of the sonication process to the pyrene fluorescence measurement was 30 s. As a control, the fluorescence of 1 µM G-actin and 1 µM actin at steady state (without sonication) was also assayed.

To deduce the concentration of G-actin from the pyrene fluorescence intensity in our samples, we proceeded as follows. At any time *t*, the fluorescence intensity can be written as *I*(*t*) = *C*_G_(*t*) *i*_G_ + *C*_F_(*t*) *i*_F_, where *C*_G_ is the G-actin concentration, *C*_F_ is the F-actin concentration, *i*_G_ is the fluorescence intensity of 1 µM G-actin, and *i*_F_ is the fluorescence intensity of 1 µM F-actin in the same conditions (same percentage of pyrene labeling, same spectrofluorimeter settings). At any time, *C*_G_(*t*) + *C*_F_(*t*) is constant and equal to the total concentration of actin in the sample. *i*_G_ is directly determined by measuring the signal from a solution of 1 µM G-actin in G-buffer. *i*_F_ is determined by measuring the signal from a solution of 1 µM actin at steady state and considering that the steady-state concentration of G-actin is 0.15 µM.

### Sample preparation for electron microscopy

G-actin stock solution was ultracentrifuged for 45 min at 80,000 × *g* before use to remove any aggregates. Actin (5 μM) was polymerized at room temperature for 1 h in F-Buffer (5 mM Tris-HCl, pH 7.8, 1 mM MgCl_2_, 0.2 mM EGTA, 0.2 mM ATP, 1 mM DTT, 50 mM KCl). To generate a high density of short actin filaments, 500 μl of poly­merized 1 µM F-actin was sonicated in F-buffer. The sonication was operated with a sonicator Vibra-Cell 75041 (20 kHz, 750 W, 20% power) equipped with a 3-mm-diameter probe. Ten pulsations of 0.1 s separated by 3.9 s rest periods were used for the sonication. Formin mDia1(SNAP-FH1FH2-DAD-His) was added in the F-actin sonicated mixture directly after the last pulsation, through a Hamilton syringe equipped with a 0.13-mm-internal-diameter needle. A formin concentration of 100 nM was used for negative stain EM experiments, and a formin concentration of 125 nM was used for cryo-EM experiments.

Twenty seconds after formin addition, 4 μl of sonicated F-actin/formin mixture was collected with a Hamilton syringe. The collected volume was directly applied to an EM grid.

For negative stain EM experiments, the solution was adsorbed 30 s on a freshly glow-discharged 300 mesh carbon-coated copper grid. Excess volume was blotted off with a filter paper (Whatman no. 1). Uranyl formiate 1% (4 μl) was transiently added before being blotted off the grid. A volume of 4 μl of rinsing uranyl formiate 1% was deposited on the grid for 30 s before drying with a paper filter (Whatman no. 1).

As shown in Supplemental Figure 1B, short filaments of a few hundred nanometers were generated. On average, 13 ends were obtained per image of ∼1 µm² (average over 1632 images). In parallel, we assessed whether generating a density of filaments by using spectrin–actin seeds would sufficiently enhance the density of short filaments at short times. However, the resulting density of short actin filaments remained low and the presence of spectrin–actin seeds that were not generating filaments induced an additional background signal that was deleterious to SPA.

For cryo-EM experiments, C-Flat R 2/2 grids were covered by graphene oxide sheets by depositing 0.2 mg/ml aqueous graphene oxide drops. The grids were used after an overnight drying step as already described (Palovcak *et al*., 2018). The graphene oxide solution concentration was estimated by spectrophotometer measurements.

The sonicated actin-F/formin mixture (4 μl) was incubated for 30 s on these freshly glow-discharged grids. The excess volume was blotted off with a filter paper (Whatman no. 1) for 3 s before the grids were plunge-frozen in liquid ethane for vitrification. These operations were carried out using an automated plunge freezing apparatus (Leica EM GP) operated at 80% humidity (Palovcak *et al*., 2018).

### Electron microscopy data collection

Negative staining EM data were collected with an FEI Tecnai G2 transmission electron microscope equipped with a LaB_6_ emission filament operating at a 200 kV acceleration voltage. Images were captured on a TVIPS F416 CMOS camera at 50,000× magnification and 1.5–2.5 μm underfocus. The corresponding pixel size was 2.13 Å per pixel. A total of 1632 images were acquired.

Cryo-EM data were collected using a Glacios 200 kV (Thermo Fisher) transmission electron microscope equipped with an FEG operating at a 200 kV acceleration voltage. Images were captured using a Falcon 3 direct detection camera at 60,000× magnification and 1–3 μm underfocus. The corresponding pixel size was 2.5 Å per pixel. Each image acquisition was performed in dose-fractionated mode with 60 frames over 2.2 s exposition time for a total dose of 60 e^–^/Å^2^ (1 e^–^/Å^2^ per frame). A total of 4223 movies were acquired.

### Estimation of the average length of actin filaments observed per image and density of formins along actin filaments

The actin filament length density observed per image was estimated to extrapolate the actin filament length imaged within the data. The actin filament length in our images was then compared with the density of formins identified along the actin filament bodies to assess the density of formin per actin filament length. The actin filament length was measured manually in 10 images and compared with the measurements resulting from a semiautomated approach on the same images (Supplemental Figure 8). An error of ∼5% was estimated between the two approaches. The semiautomated approach was applied to 32 images with a mean length of actin filament of ∼4 μm per image. This measurement extrapolated to all the images used for the picking of formins along actin filaments (632 images) led to a total length of actin filament imaged of ∼2566 μm. This length compared with the 717 particles clearly identified as formins encircling the actin filament body led to an estimation of ∼0.3 formin per micrometer of actin filament.

### Two-dimensional data processing

From the 1632 micrographs obtained by negative staining EM, 20,919 actin filament ends were handpicked using EMAN2 (Ludtke, 2016) software and extracted with a square box size of 180 pixels^2^ (2.13 Å/pixel). From 632 of these micrographs, 2013 formin-like patterns were handpicked along the actin filament cores.

A protocol was adapted to the specificity of our data. Dedicated scripts were generated within SPIDER (Frank *et al.*, 1996; Shaikh *et al.*, 2008) software. In the first round of alignment, all particles were normalized using a circular mask that roughly eclipses the signal associated with the proteins. In the second SPIDER alignment step, these normalized particles were rotationally aligned and a new normalization step was performed using a rectangular mask that more accurately eclipses the signal of the observed filaments. In a third SPIDER alignment step, a principal component analysis was performed on the rotationally aligned particles. The purpose of this step was to generate reference averages associated with a high signal-to-noise ratio that sample the various global patterns of the extracted particles. The reference averages thus generated were aligned with each other and used for a multireference alignment of the particles corresponding to the fourth SPIDER alignment step. The main purpose of this fourth step was to optimize the orientation of each particle. In this operation, a low-pass filter was applied to the particles and the reference averages to prioritize the global orientation of each particle. At this stage, a small adjustment range was allowed for the translational alignment. In a fifth SPIDER alignment step, a new principal component analysis was performed through a mask focused on the tip of the extracted filament ends or focused on the central part of the actin core filament containing a potential formin dimer. The purpose of this step was to generate reference averages representative of the structural variability and the variability in the positions of the tips. Once again, the reference averages generated were aligned with each other and used for a multireference alignment of the particles corresponding to a sixth SPIDER alignment step. The purpose of this last multireference alignment was to fine-tune the respective positions of the ends in the first case or of the formin-like pattern along the actin filament core in the second case.

Regarding the actin filament ends, the binding of FH2 domains to the actin filament tips generates asymmetrical 2D patterns with two possible configurations when the FH2 domains are observed through their main axis. The first FH2 domain can appear on either side of the observed actin filament tip, with the second FH2 domain on the corresponding opposite side. Both images of a capped filament observed from one side or the opposite side can match with these two configurations. While these two images contain the same 2D structural information, their opposing asymmetric patterns require the application of a symmetry operation along the filament axis to be correctly aligned.

In line with this observation, for each of the particles considered, the alignment score of its mirror particle (symmetry applied along the filament axis) with the reference was also evaluated. In this step, either the raw particle or its mirror particle was kept by selecting the one with the highest alignment score. On this occasion, all the references have been manually oriented in a given direction. This step allows the use of a more focused asymmetric mask around the reference averages and brings together a larger number of particles in the same classes, thus improving the associated signal-to-noise ratios. Given that the number of formin-capped ends that could be identified in the data set will be a minority, this step appears to be important to strengthen their signal.

In the seventh and last SPIDER analysis step, a principal components analysis was performed to roughly distinguish between formin-bound and bare actin filaments. The more homogeneous data sets considered to be formin bound were then subjected to a second round of 2D SPIDER analysis to benefit this time from the preponderant signal of the formins in the new particle subselection.

These aligned data sets were subjected to classical 3D analysis under RELION (Bharat and Scheres, 2016) v3 in order to benefit from the Bayesian statistical framework and thus retain only the most consistent particles. Finally, 3694 formin-bound filament ends corresponding to the “open state,” 977 formin-bound filament ends corresponding to the “closed state,” and 717 formin-bound filament cores were determined by RELION 3D classifications.

The 2D classification focused on the first FH2 domain orientation variability among the actin filament ends bound by formin in the “open state” was performed with SPIDER. From 3804 particles, four classes were generated with, respectively, 956 particles (25%), 1092 particles (29%), 651 particles (17%), and 1104 particles (29%). Angle measurements associated with the first FH2 domain orientation in each of the four classes were carried out using ImageJ. Before each measurement, the class average observed through its focused mask was thresholded to distinguish background versus protein density. The edge delimiting background and protein density was calculated with the “Find Edges” ImageJ function. This delimitation was refined with the “Skeletonize” ImageJ function. After that, the angle formed by the main axis of the actin filament and the relative orientation of the first FH2 domain was measured.

Movies acquired by cryo-EM were processed by the MotionCor2 program to correct electron beam–induced sample motion. Contrast transferred functions (CTF) from motion-corrected micrographs were estimated using the program CTFFIND4. Of 4233 corrected micrographs, 3405 were retained after eliminating images showing too much drift (broken membrane near the imaged region), aggregates of graphene oxide sheets or proteins, or poor confidence of CTF fitting estimation.

A total of 12,112 actin filament ends were handpicked within EMAN2 software and extracted with a square box size of 154 pixels^2^ (2.5 Å/pixel). From this data set and using RELION 2D classification, at least 1548 particles could be identified as actin filament ends bound by a formin dimer.

### Three-dimensional data processing

Structural models of a formin-bound barbed end in its “open state” or his “closed state” and of a formin encircling the actin filament core were generated within UCSF Chimera based on FH2-actin atomic structure (PDB: 1Y64) and actin filament atomic structure (PDB: 5OOE). These low-pass-filtered (50 Å) structural models were used as multireference models for RELION 3D classifications performed on the data sets previously determined by 2D analysis. For each data subset identified after RELION 3D classification, a crude cylinder displaying the adapted dimensions could be used as reference to generate a coherent 3D reconstruction.

After RELION 3D classifications, 3694 particles corresponding to formin-bound filament ends in their “open state” were selected and subjected to a 3D refinement, leading to a final 3D envelope at a resolution of 26 Å.

After RELION 3D classification, 977 particles corresponding to formin-bound filament ends in their “closed state” were selected and subjected to a 3D refinement, leading to a final 3D envelope at a resolution of 27 Å.

From 2013 particles corresponding to a formin dimer encircling an actin filament core, 717 particles were selected after RELION 2D classification and subjected to a 3D refinement, leading to a final 3D envelope at a resolution of 30 Å.

For cryo-EM analysis, 2373 particles were selected through RELION 3D classification, leading to a final 3D envelope at a resolution of 30 Å.

In each 3D envelope generated, the corresponding atomic model was fitted using the UCSF Chimera function “Fit in map” by allowing sequential fitting of independent domain groups. Spurious noise from EM densities was hidden with the “hide dust” command in UCSF Chimera to facilitate readability.

## Abbreviations used:

ABR: actin binding region
ATP: adenosine Tri-phosphate
Cryo-EM: cryo-electron microscopy
2D: two-dimensional
DD: dimerization domain
DID: diaphanous inhibitory domain
DTT: dithiothreitol
EGTA: ethylene glycol tetraacetic acid
EM: electron microscopy
FEG: field emission gun
FH2: formin homology domain 2
GBD: gtpase binding domain
KCl: potassium chloride
MgCl _2_: magnesium chloride
SPA: single particle analysis.
